# Supplementary material for: The association between human herpesvirus infections and stroke: a systematic review protocol
Source: BMJ Open. 2017 May 29;7(5):e016427. doi: 10.1136/bmjopen-2017-016427 (PMC5726106; doi:10.1136/bmjopen-2017-016427)
Supplement: Supplementary data [file bmjopen-2017-016427supp001.pdf]

1 exp Herpes simplex
2 exp Herpes simplex virus vaccines
3 exp encephalitis, herpes simplex
4 exp Herpesvirus 1, Human
5 cold sore$.ti,ab.
6 exp Herpesvirus 2, Human
7 (genit$ herpes$ or genit$ sores).ti,ab.
8 exp Chickenpox
9 exp Chickenpox vaccine
10 exp Herpes zoster
11 exp Neuralgia, postherpetic
12 exp Herpesvirus 3, Human
13 exp Encephalitis, varicella zoster
14 (varicella or chickenpox or chicken pox or shingles or VZV or zoster).ti,ab.
15 exp Cytomegalovirus
16 exp Cytomegalovirus vaccines
17 exp Cytomegalovirus infections
18 (CMV or cytomegalovirus).ti,ab.
19 exp Herpesvirus 6, Human
20 Roseolovirus Infections/
21 Exanthema Subitum/
(B lymphotropic virus$ or roseola or sixth disease or exanthema subitum or exanthem criticum or Roseolovirus or pseudorubella or
22 three?day fever).ti,ab.
23 exp Herpesvirus 7, Human
24 exp Epstein-Barr virus infections
25 exp Epstein-Barr virus
26 exp Herpesvirus 4, Human
27 (EBV or epstein-barr or burkitt adj5 lymphoma$ or glandular fever or infectious mono$ or mononucleosis or hair$ leukoplak$ or OHL).ti,a
28 exp Herpesvirus 8, Human
29 Sarcoma, Kaposi/
30 Lymphoma, Primary Effusion/
31 (kaposi$ sarcoma$ or Primary effusion adj2 lymphoma$ or body cavity adj2 lymphoma$).ti,ab.
((HHV adj1 ("1" or "2" or "3" or "4" or "5" or "6" or "7" or "8")) or (HHV?1 or HHV?2 or HHV?3 or HHV?4 or HHV?5 or HHV?6 or
32 HHV?7 or HHV?8)).ti,ab.
33 (HSV?1 or HSV 1 or HSV?2 or HSV 2).ti,ab.
34 herpes$.ti, ab.
35 exp acyclovir
36 ganciclovir/ or foscarnet/ or Idoxuridine/ or Trifluridine/
(ac?clovir or Zovirax or valac?clovir or valtrex or famc?clovir or famvir or penc?clovir or ganc?clovir or cidofovir or foscarnet$ or
37 valganc?clovir or lubocavir or brivudin or Docosanol or Sorivudine or Idoxuridine or Trifluridine).ti,ab
38 or 1/37
39 exp stroke/
40 exp "Intracranial Embolism and Thrombosis"/
41 exp intracranial hemorrhages/
42 aneurysm, ruptured/ and exp brain/
43 Ischemic Attack, Transient/
44 (stroke or cva or (cerebrovasc$ AND (disease or event or accident or attack or injury))).ti,ab.
((brain$ or cerebr$ or cerebell$ or cortical or vertebrobasilar or hemispher$ or intracran$ or intracerebral or infratentorial or
45 supratentorial or MCA or anterior circulation or posterior circulation or basal ganglia) adj5 (isch?emi$ or infarct$)).ti,ab.
46 (((lacunar or cortical) adj5 infarct$)).ti,ab.
47 ((intracran$ or intracerebral) adj3 (thrombo$ or thrombus$ or embol$)).ti,ab.
48 SAH.ti,ab.
((brain$ or cerebr$ or cerebell$ or intracerebral or intracran$ or parenchymal or intraventricular or infratentorial or supratentorial or
basal gangli$ or subarachnoid or putaminal or putamen or posterior fossa) adj5 (haemorrhage$ or hemorrhage$ or bleed$ or rupture$
49 adj3 aneurysm)).ti,ab.
50 ((tia$1 or transi$ adj3 (isch?emia$ attack or brain isch?emia$ or cerebral isch?emia$ or CVA$ or cerebral vasc$ or cerebrovasc$)).ti,ab.
51 or 39/50
52 38 and 51

TRIAL MODE - a valid license will remove this message. See the keywords property of this PDF for more information.
