# Supplementary material for: The association between human herpesvirus infections and stroke: a systematic review protocol
Source: BMJ Open. 2017 May 29;7(5):e016427. doi: 10.1136/bmjopen-2017-016427 (PMC5726106; doi:10.1136/bmjopen-2017-016427)
Supplement: Author's manuscript [file bmjopen-2017-016427.draft_revisions.pdf]

BMJ Open

The association between human herpesvirus infections and stroke: a systematic review protocol

Journal:	*BMJ Open*
Manuscript ID	bmjopen-2017-016427
Article Type:	Protocol
Date Submitted by the Author:	13-Feb-2017
Complete List of Authors:	Forbes, Harriet; LSHTM, NCDE<br>Benjamin, Laura; University of Liverpool Institute of Infection and Global Health<br>Breuer, Judith; University College London<br>Brown, Martin; UCL, Neurology<br>Langan, Sinead; London School of Hygiene and Tropical Medicine<br>Minassian, Caroline ; London School of Hygiene and Tropical Medicine, Faculty of Epidemiology & Population Health<br>Smeeth, Liam; London School of Hygiene and Tropical Medicine, Epidemiology and Population Health<br>Thomas, Sara; London School of Hygiene & Tropical Medicine, Faculty of Epidemiology & Population Health<br>Warren-Gash, Charlotte; London School of Hygiene and Tropical Medicine, Faculty of Epidemiology & Population Health
**Primary Subject Heading**:	Epidemiology
Secondary Subject Heading:	Cardiovascular medicine, Epidemiology, Infectious diseases, Neurology
Keywords:	Stroke < NEUROLOGY, INFECTIOUS DISEASES, EPIDEMIOLOGY, Herpesviruses

SCHOLARONE™ Manuscripts

For peer review only - http://bmjopen.bmj.com/site/about/guidelines.xhtml

The association between human herpesvirus infections and stroke: a systematic review protocol

Harriet J Forbes[1], Laura Benjamin[2,4], Judy Breuer[3], Martin M Brown[4], Sinéad M Langan[1], Caroline Minassian[1], Liam Smeeth[1], Sara L Thomas[1], Charlotte Warren-Gash[1]

Affiliations

[1]Faculty of Epidemiology & Population Health, London School of Hygiene and Tropical Medicine, London, UK

[2]Institute of Infection and Global Health, University of Liverpool, Liverpool, UK

[3]UCL Division of Infection & Immunity, UCL, London, UK

[4] Stroke Research Centre, Department of Brain Repair and Rehabilitation, UCL Institute of Neurology, University College London, UK

Corresponding author: Harriet Forbes. Email: harriet.forbes@lshtm.ac.uk. Tel: 020 7927 2174

Word count: aim 2491

For peer review only - http://bmjopen.bmj.com/site/about/guidelines.xhtml

For peer review only

ABSTRACT (<300 words)

Introduction: After primary infection, human herpesviruses establish latency and persist lifelong. Periodic virus reactivation can lead to serious inflammatory complications. Recent research suggests that herpesvirus reactivation may also be linked to acute stroke. An improved understanding of this relationship is vital to inform public health prevention strategies. We will review the evidence regarding the role of human herpesviruses in triggering stroke.

Methods and analysis: A systematic literature review of published and grey literature studies with a human herpesvirus (infection or reactivation) as an exposure and stroke as an outcome will be carried out. Randomised controlled trials, cohort, case control, case crossover and self-controlled case series designs will be eligible; no restrictions will be placed on publication status, language and geographical or healthcare setting. The Cochrane Central Register of Controlled Trials, Embase, Global Health, Medline, Scopus and Web of Science will be searched from dates of inception to January 2017. A pre-specified search strategy of medical subject headings and free text terms (in the title and abstract) for human herpesviruses AND stroke will be used. Two reviewers will independently screen titles and abstracts for eligible studies, followed by full-text screening. The reviewers will then extract data from the eligible studies using standardised, pilot-tested tables and assess risk of bias in individual studies, in line with the Cochrane Collaboration approach. The data will be synthesised in a narrative format, and meta-analyses considered where there are sufficient data. Quality of evidence will be assessed in line with the GRADE approach.

Ethics and dissemination: As this is a systematic review, ethical approval is not required. The results will be submitted for peer-review publication and presented at national conferences. A lay and short summary will be disseminated on appropriate webpages.

PROSPERO registration number: CRD42017054502

For peer review only - http://bmjopen.bmj.com/site/about/guidelines.xhtml

Strengths and limitations of this study

- This systematic review will comprehensively evaluate studies of both infection with, and reactivation of, all eight human herpesviruses and the risk of subsequent stroke.
- An improved understanding of this relationship may help to inform public health stroke prevention strategies.
- We will use the GRADE system to ascertain the strength of the evidence base for each human herpesvirus and the risk of stroke, and report data in a 'Summary of Findings' table.
- Included studies may have substantially different methodologies, which could limit our ability to draw reliable conclusions from the existing evidence base.

For peer review only

For peer review only - http://bmjopen.bmj.com/site/about/guidelines.xhtml

INTRODUCTION

Rationale

Stroke is the world's second most common cause of death[1] and the leading cause of complex disability in the UK.[2] Age is the most important risk factor for stroke.[2] Although the incidence of stroke is falling, the ageing population means that the burden of disease due to stroke (including disability, illness and premature death) is projected to double worldwide by 2030.[1] While traditional risk factors for stroke are well-characterised,[3] a growing literature highlights the role of non-traditional transient factors such as infections as vascular triggers.[4]

The herpesviruses are a family of common persistent viruses that may reactivate periodically from latency to cause substantial morbidity through inducing a range of inflammatory effects. Reactivation of varicella zoster virus (VZV) causes an acute shingles (or herpes zoster) episode, resulting in tissue damage and inflammation, and reactivation of herpes simplex virus types 1 and 2 can lead to corneal blindness and meningoencephalitis.[5]

Recent population studies have shown a short-term increase in the risk of stroke in months following infection with or reactivation of VZV.[6-8] These data come from powerful self-controlled case series studies using primary care electronic health records from both the UK and US, and are corroborated by several prospective cohort studies using data from Asian and European populations.[9-12] The effects of other members of the herpesvirus family on vascular events is less clear, although cytomegalovirus (CMV) is hypothesised to modulate stroke risk, especially among immunocompromised populations.[13]

Two recently published reviews investigated the evidence for short and long-term risks of stroke after herpes zoster.[14,15] One showed a risk ratio of 1.36 (95% C.I. 1.10-1.67) for the association

For peer review only - http://bmjopen.bmj.com/site/about/guidelines.xhtml

For peer review only

between herpes zoster and stroke pooled across six cohort studies.[15] The other meta-analysis used data from eight studies to show a gradient of stroke risk decreasing from 2.36 (95% C.I. 2.17-2.56) in the first two weeks after herpes zoster to 1.56 (95% C.I. 1.46-1.66) at one month, 1.17 (95% C.I. 1.13-1.22) at one year and 1.09 (95% C.I. 1.02-1.16) after one year.[14] These studies were limited to clinical VZV reactivation, and did not investigate risks associated with initial infection, or sub-clinical reactivation. Although one of the reviews presented results for some sub-group analyses,[15] the exclusion of self-controlled case series studies limited power to detect effects on population sub-groups or stroke sub-types.

To extend the work carried out in previous reviews, we will comprehensively review studies of both infection with and reactivation of all eight human herpesviruses and risk of stroke. In pre-specified sub-group analyses we will assess whether the effects of herpesviruses on stroke differ among population sub-groups e.g. stratified by age group and immune status, at different time periods after infection or reactivation and on stroke sub-types. We will also assess whether there is any evidence that stroke risk is modulated by preventing or treating herpesvirus infection or reactivation using vaccines or antiviral agents such as acyclovir.

Objectives

The primary objective of the planned systematic review is to investigate whether patients with primary infection, or reactivation of, human herpesviruses are at increased risk of stroke, compared to those without (or with latent) human herpesviruses.

The review will also assess the following secondary research questions:

1. Does preventing or treating human herpesviruses attenuate the risk of stroke?
2. Does the association between human herpesviruses and stroke vary by population characteristics (such as age and other common vascular risk factors)?

For peer review only - http://bmjopen.bmj.com/site/about/guidelines.xhtml

3. Does primary infection with or reactivation of human herpesviruses increase the risk of subtypes of stroke or transient ischaemic attack (TIA)?

These objectives will be addressed through a comprehensive review targeting all analytical epidemiological studies in humans of any age.

METHODS

This protocol follows the Preferred Reporting Items for Systematic Reviews and Meta Analyses Protocols (PRISMA-P) guidelines.[16]

Eligibility criteria

Study designs and characteristics: We will include studies using randomised controlled trials, cohort, case control, case crossover and self-controlled case series designs, reporting an effect estimate or the data that allow its calculation. We will exclude cross-sectional studies, ecological studies, case series, case reports and reviews. Studies from any time period, of any publication status, reported in any language and conducted in any geographical and healthcare setting (including in-patient, out-patient, primary care and community settings) will be considered.

Participants: Eligible studies will include human participants of any age and any immunosuppression status. Animal studies will not be included.

Exposure: The exposures of interest are infection with or reactivation of the eight human herpesviruses: specifically, herpes simplex virus types 1 and 2, varicella zoster virus (VZV), Epstein-Barr virus (EBV), cytomegalovirus (CMV), human herpesvirus 6, human herpesvirus 7, and human herpesvirus 8. Studies involving an exposed participant group whose members self-report infection

For peer review only

or reactivation with a human herpes virus, or who have a confirmed diagnosis, either through clinical or laboratory criteria, will be included in the review. We will also include vaccination against herpesviruses (e.g. zostavax vaccine) and treatment for herpesviruses (e.g. antivirals such as acyclovir) in order to investigate whether preventing or treating human herpesviruses attenuate the risk of stroke (a secondary research question).

Comparators: Eligible studies must include a comparator group who are unexposed, that is people (or person time for self-controlled case series designs) without herpesvirus infections or with latent herpesvirus infections.

Outcomes: Studies will be included in the review if the primary outcome was any stroke, clinically diagnosed or self-reported, and the patient's first ever or subsequent stroke. For studies meeting the inclusion criteria, we will additionally assess the following secondary outcomes: TIA (a transient episode of neurological dysfunction caused by focal brain, spinal cord, or retinal ischemia without acute infarction)[17] and subtypes of stroke (ischaemic versus haemorrhagic). Most strokes (approximately 85%)[2] are ischaemic (an episode of neurological dysfunction caused by focal, cerebral, spinal or retinal infarction)[17], compared to haemorrhagic (neurological dysfunction caused by a focal collection of blood within or on the surface of the brain).[17]

Eligibility criteria may be further developed, in an iterative process, after preliminary searches.

Information sources

The following databases will be searched for relevant articles, from dates of inception to January 2017; Cochrane Central Register of Controlled Trials (The Cochrane Library), Embase, Global Health, Medline, Scopus and Web of Science. Additional sources which will be searched include clinical trials

For peer review only - http://bmjopen.bmj.com/site/about/guidelines.xhtml

registers (such as ClinicalTrials.gov) and grey literature including the New York Academy of Medicine Grey Literature Report (www.greylit.org) and the Electronic Theses Online Service through the British Library (http://ethos.bl.uk). PROSPERO will also be periodically checked for ongoing and completed systematic reviews concerning stroke and herpesviruses.

Search strategy

The search strategy will consist of searching medical subject heading (MeSH) terms and free text (in the title and abstract) for the concepts 'human herpesviruses' and 'stroke' (combined with the Boolean logic operator AND). The provisional search terms have been developed for the database MEDLINE and will be transcribed into appropriate search terms for the other information sources. The list of proposed search terms has been reviewed by all collaborators, including those with medical knowledge of the subject area, and necessary adjustments were made. The provisional search terms for MEDLINE are listed in the appendix.

We will review the reference lists of eligible articles and relevant reviews to identify additional papers not indexed in the databases searched.

Study records

Data management: Citations identified from the literature search will be downloaded into EndNote version X7.5 and duplicate records removed by one author.

Selection process: Two researchers (HF and CWG) will review all titles and abstracts in parallel to select studies for inclusion. To reduce the risk of missing potentially relevant studies, a deliberately lenient approach will be adopted for this first level of screening. Both authors will then obtain full-text articles for studies deemed to potentially meet the review criteria. Reasons for rejection of articles during the full-text screening process will be noted, according to a hierarchical list (ineligible

For peer review only

study design, wrong exposure, wrong outcome, insufficient information to calculate an effect estimate). Any discrepancies will be discussed by HF and CWG and consultation with a third reviewer (CM) will be carried out where necessary.

Data extraction: Information will be extracted from each study selected for review. Data extraction tables will be piloted by two authors (HF and CWG) for three studies and changes to the extraction tables made as required. Any discrepancies between the two authors will be discussed, and consultation with a third author (CM) carried out if required. Data will be extracted for each remaining study by a single author (HF). Consideration will be given to contacting corresponding authors for any missing information or clarification on unclear information, using a standard email template.

Data items

Data will be extracted using a standardised template. We will use the PICOS[18] (Population, Intervention, Comparator, Outcomes and Study design) framework, originally devised to formulate a research question, as a basis to develop data extraction criteria. As this is an aetiological study, "exposure" will replace "intervention" and "study characteristics" will replace "study design". Data items on the following five domains will be extracted;

1. *Population:* characteristics of the study population (e.g. mean/median age, ethnic distribution, immune status), inclusion and exclusion criteria;
2. *Exposure:* definition and identification of human herpesvirus exposure, number of exposed subjects;
3. *Comparators:* definition and identification of unexposed individuals, number of unexposed subjects;

For peer review only - http://bmjopen.bmj.com/site/about/guidelines.xhtml

4. *Outcomes:* definition and identification of primary (stroke) and secondary outcomes (stroke subtypes or TIA), number of subjects with outcome;
5. *Study characteristics:* authors, publication year, setting/source of participants, design, methods of recruitment and sampling, period of study, length of follow-up time (if relevant), aims and objectives.

In terms of the study results, unadjusted and fully adjusted effect estimates for the association between herpesviruses and stroke will be recorded. Details of the confounders measured and adjusted for will also be noted. Results of any additional stratified analyses will also be recorded. Where possible, results from additional subgroup analyses with evidence regarding our non-primary objectives will also be recorded, for example the association between herpesviruses and the secondary outcomes (stroke subtype or TIA).

Outcomes and prioritization

The primary clinical outcome of interest is the first record of stroke following infection with or reactivation of a human herpesvirus. Where studies report several results for risk of stroke following herpesvirus exposure we will prioritise: stroke diagnosed objectively (e.g. through neuroimaging) or clinically, for example meeting the American Heart Association/American Stroke Association definition;[17] outcomes reported for the whole cohort (rather than subsets of the cohort, whose association between the herpesvirus and stroke may differ) and fully adjusted estimates of effect (rather than crude estimates). We will also extract data on the following secondary outcomes, where they are reported: TIA and subtypes of stroke (i.e. ischaemic versus haemorrhagic). Data extraction for these additional outcomes will be prioritised in the same way as the primary outcome. Studies in which exposures were recorded prior to outcomes will be prioritized when considering the overall quality of included studies.

For peer review only - http://bmjopen.bmj.com/site/about/guidelines.xhtml

For peer review only

Risk of bias in individual studies

Two authors (HF and CWG) will independently evaluate the risk of bias in three studies, and any discrepancies will be discussed and our third reviewer (CM) consulted if necessary. HF will then carry out the risk of bias assessment for the remaining studies. We will consider a series of relevant areas of bias (or domains) for each individual study, in line with the Cochrane Collaborations risk of bias approach.[19-21] For observational studies, domains will include bias due to: 1) confounding; 2) selection of participants; and 3) differential and non-differential misclassification of variables (exposures, outcomes and covariates) 4) bias due to missing data. For randomised controlled trials, domains will include random sequence generation, allocation concealment, blinding, completeness of outcome data and selective reporting. Each domain will be classified as either 'high risk' (if criterion are inadequately addressed), 'low risk' (if criterion are adequately addressed) or 'unclear risk' (if information is insufficient to formulate a judgement). A summary risk of bias table will be produced, with an additional table briefly justifying each judgement included in the appendix.

Data synthesis and meta-bias(es)

We will use a narrative synthesis, in which studies are grouped by each specific herpesvirus exposure, to summarise the evidence for the association between the herpesvirus and our primary outcome (stroke). If there are sufficient data in the selected studies our narrative synthesis will also describe subgroup analyses, relevant to our secondary research questions. These include: 1) the effect of herpesviruses on stroke, according to whether patients were vaccinated (e.g. with the chickenpox vaccine or the herpes zoster vaccine) or received antiviral treatment against herpesviruses; 2) the effect of herpesviruses on stroke for population characteristics, such as age strata and other common vascular risk factors; and 3) the effect of herpesviruses on the secondary

For peer review only - http://bmjopen.bmj.com/site/about/guidelines.xhtml

outcomes TIA and stroke subtype (such as ischemic stroke or haemorrhagic stroke, timing of stroke, first or subsequent stroke).

If there are at least two eligible studies assessing the same herpesvirus as a risk factor for our primary (stroke) or secondary (TIA or stroke type) outcomes, which are sufficiently homogenous in terms of design, study population and outcome, we will consider conducting a meta-analysis to calculate a pooled effect estimate. The choice of whether to conduct a meta-analysis, and which model to adopt (fixed or random effects) will be guided by the level of statistical heterogeneity assessed using the Cochrane Q statistic and the I^2 statistic. An $I^2<50\%$ will be used as a threshold to indicate moderate heterogeneity and potential to use of a random effects model, if there is overall consistency in the direction of effect. We will investigate sources of heterogeneity by removing studies at high risk of bias and comparing summary estimates from different study-level methodological and clinical characteristics (such as stroke definition, study design and age of the study population), using meta-regression where appropriate. Publication bias will be considered using funnel plots. All of the statistical analysis will be performed using STATA version 14.0.

Confidence in cumulative evidence

The Grading of Recommendations, Assessment, Development and Evaluation (GRADE)[22] approach will be used to summarise the quality of cumulative evidence for each herpesvirus on our outcomes, stratified by exposure definition and population characteristics. In addition to the risk of bias domains outlined earlier, we will also assess inconsistency between studies, indirectness, imprecision of estimates and publication bias (using a funnel plot) as outlined in the GRADE approach.[23] The strength of evidence will be categorised as 'high', 'moderate' or 'low/very low', with observational studies starting as low quality evidence, but upgraded to moderate or even high

For peer review only - http://bmjopen.bmj.com/site/about/guidelines.xhtml

quality in the presence of factors that increase confidence in the estimated effect data (for example having a large magnitude of effect, evidence of a dose-response). These judgements will be presented in a 'Summary of Findings' table.

ETHICS AND DISSEMINATION

As this is a systematic review ethics approval is not required. The results will be submitted for peer-review publication and presented at national and international conferences. A lay and short summary will be disseminated on appropriate webpages. Important protocol amendments will be documented with a justification for deviating from the original protocol, and summarised in a protocol addendum and in the final published review.

References

1. Feigin VL, Forouzanfar MH, Krishnamurthi R, et al. Global and regional burden of stroke during 1990-2010: findings from the Global Burden of Disease Study 2010. Lancet 2014: 383(9913): 245-54.
2. Stroke Association. *State of the nation: Stroke Statistics*. 2016 [Cited 11 Jan 2017]; Available from: https://www.stroke.org.uk/resources/state-nation-stroke-statistics.
3. O'Donnell MJ, Xavier D, Liu L, et al. Risk factors for ischaemic and intracerebral haemorrhagic stroke in 22 countries (the INTERSTROKE study): a case-control study. The Lancet 376(9735): 112-123.
4. Esenwa CC and Elkind MS. Inflammatory risk factors, biomarkers and associated therapy in ischaemic stroke. Nature Reviews Neurology 2016: 12(10): 594-604.
5. Steiner I, Kennedy PG, and Pachner AR. The neurotropic herpes viruses: herpes simplex and varicella-zoster. Lancet Neurol 2007: 6(11): 1015-28.
6. Thomas SL, Minassian C, Ganesan V, et al. Chickenpox and Risk of Stroke: A Self-controlled Case Series Analysis. Clinical Infectious Diseases 2014: 58(1): 61-68.
7. Langan S, Minassian C, Smeeth L, and Thomas S. Risk of stroke following herpes zoster: a self-controlled case-series study. Journal of Investigative Dermatology 2014: 134: S39-S39.
8. Minassian C, Thomas SL, Smeeth L, et al. Acute Cardiovascular Events after Herpes Zoster: A Self-Controlled Case Series Analysis in Vaccinated and Unvaccinated Older Residents of the United States. Plos Medicine 2015: 12(12).
9. Kang JH, Ho JD, Chen YH, and Lin HC. Increased Risk of Stroke After a Herpes Zoster Attack A Population-Based Follow-Up Study. Stroke 2009: 40(11): 3443-3448.
10. Lin HC, Chien CW, and Ho JD. Herpes zoster ophthalmicus and the risk of stroke A population-based follow-up study. Neurology 2010: 74(10): 792-797.

For peer review only - http://bmjopen.bmj.com/site/about/guidelines.xhtml

11. Sundstrom K, Weibull CE, Soderberg-Lofdal K, et al. Incidence of herpes zoster and associated events including stroke-a population-based cohort study. Bmc Infectious Diseases 2015: 15.
12. Sreenivasan N, Basit S, Wohlfahrt J, et al. The Short- and Long-Term Risk of Stroke after Herpes Zoster - A Nationwide Population-Based Cohort Study. Plos One 2013: 8(7).
13. Yen YF, Chen M, Jen I, et al. Association of HIV and Opportunistic Infections with Incident Stroke: A Nationwide Population-based Cohort Study in Taiwan. J Acquir Immune Defic Syndr 2016.
14. Liu XC, Guan YM, Hou L, et al. The Short- and Long-Term Risk of Stroke after Herpes Zoster: A Meta-Analysis. Plos One 2016: 11(10).
15. Yang SY, Li HX, Yi XH, et al. Risk of Stroke in Patients with Herpes Zoster: A Systematic Review and Meta-Analysis. J Stroke Cerebrovasc Dis 2016.
16. Shamseer L, Moher D, Clarke M, et al. Preferred reporting items for systematic review and meta-analysis protocols (PRISMA-P) 2015: elaboration and explanation. BMJ 2015: 349: g7647.
17. Sacco RL, Kasner SE, Broderick JP, et al. An Updated Definition of Stroke for the 21st Century. A Statement for Healthcare Professionals From the American Heart Association/American Stroke Association 2013: 44(7): 2064-2089.
18. Higgins J and Green S. *Cochrane Handbook for Systematic Reviews of Interventions Version 5.1.0 [updated March 2011]. 6.4.2 Structure of a search strategy.* 2011 [Cited; Available from: www.handbook.cochrane.org.
19. Sterne JA, Hernan MA, Reeves BC, et al. ROBINS-I: a tool for assessing risk of bias in non-randomised studies of interventions. BMJ 2016: 355: i4919.
20. Higgins JP, Altman DG, Gotzsche PC, et al. The Cochrane Collaboration's tool for assessing risk of bias in randomised trials. BMJ 2011: 343: d5928.
21. Sterne JA, Hernán MA, Reeves BC, et al. ROBINS-I: a tool for assessing risk of bias in non-randomised studies of interventions. BMJ 2016: 355.
22. Atkins D, Best D, Briss PA, et al. Grading quality of evidence and strength of recommendations. British Medical Journal 2004: 328(7454): 1490-1494.
23. Balshem H, Helfand M, Schunemann HJ, et al. GRADE guidelines: 3. Rating the quality of evidence. Journal of Clinical Epidemiology 2011: 64(4): 401-406.

Authors' contributions

HF contributed to the design of the study, drafted the methods and analysis and revised the protocol following author comments; LB contributed to the design of the study and revised the paper critically; JB contributed to the conception and design of the study and revised the paper critically; MB contributed to the design of the study and revised the paper critically; SML contributed to the design of the study and revised the paper critically; CM contributed to the design of the study and revised the paper critically; LS contributed to the conception and design of the study and revised the paper critically; SLT contributed to the design of the study and revised the paper critically; CWG conceived and designed the study, drafted the introduction and made critical comments on the protocol. All authors approved the final version of the protocol.

For peer review only

Funding statement

Funded by a Wellcome Trust Intermediate Clinical Fellowship to Dr C. Warren-Gash (201440/Z/16/Z). The funder played no role in developing the protocol. JB receives funding from the NIHR UCL/UCLH . BRC. SML is funded by a Wellcome Trust Senior Research Fellowship in Clinical Science (205039/Z/16/Z). CM is supported by a Wellcome Trust Senior Fellowship in Clinical Science (to LS, grant number: 098504/Z/12/Z). LB is supported by NIHR Clinical Lectureship funding. LS is supported by a Senior Clinical Fellowship from Wellcome. MMB's Chair in Stroke Medicine is supported by the Reta Lila Weston Trust for Medical Research. Part of this work was undertaken at University College London, which received a proportion of funding from the UK Department of Health's National Institute for Health Research Biomedical Research Centres funding scheme.

Competing interests statement

HF - none; LB – none; JB – none; MB - none; SML – none; CM – none; LS – none; SLT – none; CWG – none.

For peer review only

1 exp Herpes simplex
2 exp Herpes simplex virus vaccines
3 exp encephalitis, herpes simplex
4 exp Herpesvirus 1, Human
5 cold sore$.ti,ab.
6 exp Herpesvirus 2, Human
7 (genit$ herpes$ or genit$ sores).ti,ab.
8 exp Chickenpox
9 exp Chickenpox vaccine
10 exp Herpes zoster
11 exp Neuralgia, postherpetic
12 exp Herpesvirus 3, Human
13 exp Encephalitis, varicella zoster
14 (varicella or chickenpox or chicken pox or shingles or VZV or zoster).ti,ab.
15 exp Cytomegalovirus
16 exp Cytomegalovirus vaccines
17 exp Cytomegalovirus infections
18 (CMV or cytomegalovirus).ti,ab.
19 exp Herpesvirus 6, Human
20 Roseolovirus Infections/
21 Exanthema Subitum/
22 (B lymphotropic virus$ or roseola or sixth disease or exanthema subitum or exanthem criticum or Roseolovirus or pseudorubella or three?day fever).ti,ab.
23 exp Herpesvirus 7, Human
24 exp Epstein-Barr virus infections
25 exp Epstein-Barr virus
26 exp Herpesvirus 4, Human
27 (EBV or epstein-barr or burkitt adj5 lymphoma$ or glandular fever or infectious mono$ or mononucleosis or hair$ leukoplak$ or OHL).ti,a
28 exp Herpesvirus 8, Human
29 Sarcoma, Kaposi/
30 Lymphoma, Primary Effusion/
31 (kaposi$ sarcoma$ or Primary effusion adj2 lymphoma$ or body cavity adj2 lymphoma$).ti,ab.
32 ((HHV adj1 ("1" or "2" or "3" or "4" or "5" or "6" or "7" or "8")) or (HHV?1 or HHV?2 or HHV?3 or HHV?4 or HHV?5 or HHV?6 or HHV?7 or HHV?8)).ti,ab.
33 (HSV?1 or HSV 1 or HSV?2 or HSV 2).ti,ab.
34 herpes$.ti, ab.
35 exp acyclovir
36 ganciclovir/ or foscarnet/ or Idoxuridine/ or Trifluridine/
37 (ac?clovir or Zovirax or valac?clovir or valtrex or famc?clovir or famvir or penc?clovir or ganc?clovir or cidofovir or foscarnet$ or valganc?clovir or lubocavir or brivudin or Docosanol or Sorivudine or Idoxuridine or Trifluridine).ti,ab
38 or 1/37
39 exp stroke/
40 exp "Intracranial Embolism and Thrombosis"/
41 exp intracranial hemorrhages/
42 aneurysm, ruptured/ and exp brain/
43 Ischemic Attack, Transient/
44 (stroke or cva or (cerebrovasc$ AND (disease or event or accident or attack or injury))).ti,ab.
45 ((brain$ or cerebr$ or cerebell$ or cortical or vertebrobasilar or hemispher$ or intracran$ or intracerebral or infratentorial or supratentorial or MCA or anterior circulation or posterior circulation or basal ganglia) adj5 (isch?emi$ or infarct$)).ti,ab.
46 (((lacunar or cortical) adj5 infarct$)).ti,ab.
47 ((intracran$ or intracerebral) adj3 (thrombo$ or thrombus$ or embol$)).ti,ab.
48 SAH.ti,ab.
49 ((brain$ or cerebr$ or cerebell$ or intracerebral or intracran$ or parenchymal or intraventricular or infratentorial or supratentorial or basal gangli$ or subarachnoid or putaminal or putamen or posterior fossa) adj5 (haemorrhage$ or hemorrhage$ or bleed$ or rupture$ adj3 aneurysm)).ti,ab.
50 ((tia$1 or transi$ adj3 (isch?emia$ attack or brain isch?emia$ or cerebral isch?emia$ or CVA$ or cerebral vasc$ or cerebrovasc$)).ti,ab.
51 or 39/50
52 38 and 51

For peer review only

TRIAL MODE - a valid license will remove this message. See the keywords property of this PDF for more information.

PRISMA-P (Preferred Reporting Items for Systematic review and Meta-Analysis Protocols) 2015 checklist: recommended items to address in a systematic review protocol*

Section and topic	Item No	Checklist item		
**ADMINISTRATIVE INFORMATION**				
Title:				
Identification	1a	Identify the report as a protocol of a systematic review	✓	P1
Update	1b	If the protocol is for an update of a previous systematic review, identify as such	-	-
Registration	2	If registered, provide the name of the registry (such as PROSPERO) and registration number	✓	P2
Authors:				
Contact	3a	Provide name, institutional affiliation, e-mail address of all protocol authors; provide physical mailing address of corresponding author	✓	P1
Contributions	3b	Describe contributions of protocol authors and identify the guarantor of the review	✓	P14
Amendments	4	If the protocol represents an amendment of a previously completed or published protocol, identify as such and list changes; otherwise, state plan for documenting important protocol amendments	-	-
Support:				
Sources	5a	Indicate sources of financial or other support for the review	✓	P15
Sponsor	5b	Provide name for the review funder and/or sponsor	✓	P15
Role of sponsor or funder	5c	Describe roles of funder(s), sponsor(s), and/or institution(s), if any, in developing the protocol	✓	P15
**INTRODUCTION**				
Rationale	6	Describe the rationale for the review in the context of what is already known	✓	P4/5
Objectives	7	Provide an explicit statement of the question(s) the review will address with reference to participants, interventions, comparators, and outcomes (PICO)	✓	P5
**METHODS**				
Eligibility criteria	8	Specify the study characteristics (such as PICO, study design, setting, time frame) and report characteristics (such as years considered, language, publication status) to be used as criteria for eligibility for the review	✓	P6/7
Information sources	9	Describe all intended information sources (such as electronic databases, contact with study authors, trial registers or other grey literature sources) with planned dates of coverage	✓	P7/8

For peer review only

Search strategy	10	Present draft of search strategy to be used for at least one electronic database, including planned limits, such that it could be repeated	✓	P8
Study records:				
Data management	11a	Describe the mechanism(s) that will be used to manage records and data throughout the review	✓	P8
Selection process	11b	State the process that will be used for selecting studies (such as two independent reviewers) through each phase of the review (that is, screening, eligibility and inclusion in meta-analysis)	✓	P8/9
Data collection process	11c	Describe planned method of extracting data from reports (such as piloting forms, done independently, in duplicate), any processes for obtaining and confirming data from investigators	✓	P9
Data items	12	List and define all variables for which data will be sought (such as PICO items, funding sources), any pre-planned data assumptions and simplifications	✓	P9/10
Outcomes and prioritization	13	List and define all outcomes for which data will be sought, including prioritization of main and additional outcomes, with rationale	✓	P10
Risk of bias in individual studies	14	Describe anticipated methods for assessing risk of bias of individual studies, including whether this will be done at the outcome or study level, or both; state how this information will be used in data synthesis	✓	P11
Data synthesis	15a	Describe criteria under which study data will be quantitatively synthesised	✓	P11/12
	15b	If data are appropriate for quantitative synthesis, describe planned summary measures, methods of handling data and methods of combining data from studies, including any planned exploration of consistency (such as I^2, Kendall's τ)	✓	P11/12
	15c	Describe any proposed additional analyses (such as sensitivity or subgroup analyses, meta-regression)	✓	P11/12
	15d	If quantitative synthesis is not appropriate, describe the type of summary planned	✓	P11/12
Meta-bias(es)	16	Specify any planned assessment of meta-bias(es) (such as publication bias across studies, selective reporting within studies)	✓	P11/12
Confidence in cumulative evidence	17	Describe how the strength of the body of evidence will be assessed (such as GRADE)	✓	P12/13

*** It is strongly recommended that this checklist be read in conjunction with the PRISMA-P Explanation and Elaboration (cite when available) for important clarification on the items. Amendments to a review protocol should be tracked and dated. The copyright for PRISMA-P (including checklist) is held by the PRISMA-P Group and is distributed under a Creative Commons Attribution Licence 4.0.**

From: Shamseer L, Moher D, Clarke M, Ghersi D, Liberati A, Petticrew M, Shekelle P, Stewart L, PRISMA-P Group. Preferred reporting items for systematic review and meta-analysis protocols (PRISMA-P) 2015: elaboration and explanation. BMJ. 2015 Jan 2;349(jan02 1):g7647.

For peer review only

BMJ Open

The association between human herpesvirus infections and stroke: a systematic review protocol

Journal:	*BMJ Open*
Manuscript ID	bmjopen-2017-016427.R1
Article Type:	Protocol
Date Submitted by the Author:	27-Mar-2017
Complete List of Authors:	Forbes, Harriet; LSHTM, NCDE<br>Benjamin, Laura; University of Liverpool Institute of Infection and Global Health<br>Breuer, Judith; University College London<br>Brown, Martin; UCL, Neurology<br>Langan, Sinead; London School of Hygiene and Tropical Medicine<br>Minassian, Caroline ; London School of Hygiene and Tropical Medicine, Faculty of Epidemiology & Population Health<br>Smeeth, Liam; London School of Hygiene and Tropical Medicine, Epidemiology and Population Health<br>Thomas, Sara; London School of Hygiene & Tropical Medicine, Faculty of Epidemiology & Population Health<br>Warren-Gash, Charlotte; London School of Hygiene and Tropical Medicine, Faculty of Epidemiology & Population Health
**Primary Subject Heading**:	Epidemiology
Secondary Subject Heading:	Cardiovascular medicine, Epidemiology, Infectious diseases, Neurology
Keywords:	Stroke < NEUROLOGY, INFECTIOUS DISEASES, EPIDEMIOLOGY, Herpesviruses

SCHOLARONE™
Manuscripts

The association between human herpesvirus infections and stroke: a systematic review protocol

Harriet J Forbes[1], Laura Benjamin[2,4], Judy Breuer[3], Martin M Brown[4], Sinéad M Langan[1], Caroline Minassian[1], Liam Smeeth[1], Sara L Thomas[1], Charlotte Warren-Gash[1]

Affiliations

[1]Faculty of Epidemiology & Population Health, London School of Hygiene and Tropical Medicine, London, UK

[2]Institute of Infection and Global Health, University of Liverpool, Liverpool, UK

[3]UCL Division of Infection & Immunity, UCL, London, UK

[4] Stroke Research Centre, Department of Brain Repair and Rehabilitation, UCL Institute of Neurology, University College London, UK

Corresponding author: Harriet Forbes. Email: harriet.forbes@lshtm.ac.uk. Tel: 020 7927 2174

Word count: aim 2491

For peer review only

For peer review only - http://bmjopen.bmj.com/site/about/guidelines.xhtml

ABSTRACT (<300 words)

Introduction: After primary infection, human herpesviruses establish latency and persist lifelong. Periodic virus reactivation can lead to serious inflammatory complications. Recent research suggests that herpesvirus reactivation may also be linked to acute stroke. An improved understanding of this relationship is vital to inform public health prevention strategies. We will review the evidence regarding the role of human herpesviruses in triggering stroke.

Methods and analysis: A systematic literature review of published and grey literature studies with a human herpesvirus (infection or reactivation) as an exposure and stroke as an outcome will be carried out. Randomised controlled trials, cohort, case control, case crossover and self-controlled case series designs will be eligible; no restrictions will be placed on publication status, language and geographical or healthcare setting. The Cochrane Central Register of Controlled Trials, Embase, Global Health, Medline, Scopus and Web of Science will be searched from dates of inception to January 2017. A pre-specified search strategy of medical subject headings and free text terms (in the title and abstract) for human herpesviruses AND stroke will be used. Two reviewers will independently screen titles and abstracts for eligible studies, followed by full-text screening. The reviewers will then extract data from the eligible studies using standardised, pilot-tested tables and assess risk of bias in individual studies, in line with the Cochrane Collaboration approach. The data will be synthesised in a narrative format, and meta-analyses considered where there are sufficient data. Quality of evidence will be assessed in line with the GRADE approach.

Ethics and dissemination: As this is a systematic review, ethical approval is not required. The results will be submitted for peer-review publication and presented at national conferences. A lay and short summary will be disseminated on appropriate webpages.

PROSPERO registration number: CRD42017054502

For peer review only - http://bmjopen.bmj.com/site/about/guidelines.xhtml

Strengths and limitations of this study

- This systematic review will comprehensively evaluate studies of both infection with, and reactivation of, all eight human herpesviruses and the risk of subsequent stroke.
- An improved understanding of this relationship may help to inform public health stroke prevention strategies.
- We will use the GRADE system to ascertain the strength of the evidence base for each human herpesvirus and the risk of stroke, and report data in a 'Summary of Findings' table.
- Included studies may have substantially different methodologies, which could limit our ability to draw reliable conclusions from the existing evidence base.

For peer review only

For peer review only - http://bmjopen.bmj.com/site/about/guidelines.xhtml

INTRODUCTION

Rationale

Stroke is the world's second most common cause of death[1] and the leading cause of complex disability in the UK.[2] Age is the most important risk factor for stroke.[2] Although the incidence of stroke is falling, the ageing population means that the burden of disease due to stroke (including disability, illness and premature death) is projected to double worldwide by 2030.[1] While traditional risk factors for stroke are well-characterised,[3] a growing literature highlights the role of non-traditional transient factors such as infections as vascular triggers.[4]

The herpesviruses are a family of common persistent viruses that may reactivate periodically from latency to cause substantial morbidity through inducing a range of inflammatory effects. Reactivation of varicella zoster virus (VZV) causes an acute shingles (or herpes zoster) episode, resulting in tissue damage and inflammation, and reactivation of herpes simplex virus types 1 and 2 can lead to corneal blindness and meningoencephalitis.[5]

Recent population studies have shown a short-term increase in the risk of stroke in months following infection with or reactivation of VZV.[6-8] These data come from powerful self-controlled case series studies using primary care electronic health records from both the UK and US, and are corroborated by several prospective cohort studies using data from Asian and European populations.[9-12] The effects of other members of the herpesvirus family on vascular events is less clear, although cytomegalovirus (CMV) is hypothesised to modulate stroke risk, especially among immunocompromised populations.[13]

Two recently published reviews investigated the evidence for short and long-term risks of stroke after herpes zoster.[14,15] One showed a risk ratio of 1.36 (95% C.I. 1.10-1.67) for the association

For peer review only - http://bmjopen.bmj.com/site/about/guidelines.xhtml

between herpes zoster and stroke pooled across six cohort studies.[15] The other meta-analysis used data from eight studies to show a gradient of stroke risk decreasing from 2.36 (95% C.I. 2.17-2.56) in the first two weeks after herpes zoster to 1.56 (95% C.I. 1.46-1.66) at one month, 1.17 (95% C.I. 1.13-1.22) at one year and 1.09 (95% C.I. 1.02-1.16) after one year.[14] These studies were limited to clinical VZV reactivation, and did not investigate risks associated with initial infection, or sub-clinical reactivation. Although one of the reviews presented results for some sub-group analyses,[15] the exclusion of self-controlled case series studies limited power to detect effects on population sub-groups or stroke sub-types.

To extend the work carried out in previous reviews, we will comprehensively review studies of both infection with and reactivation of all eight human herpesviruses and risk of stroke. In pre-specified sub-group analyses we will assess whether the effects of herpesviruses on stroke differ among population sub-groups e.g. stratified by age group and immune status, at different time periods after infection or reactivation and on stroke sub-types. We will also assess whether there is any evidence that stroke risk is modulated by preventing or treating herpesvirus infection or reactivation using vaccines or antiviral agents such as acyclovir.

Objectives

The primary objective of the planned systematic review is to investigate whether patients with primary infection, or reactivation of, human herpesviruses are at increased risk of stroke, compared to those without (or with latent) human herpesviruses.

The review will also assess the following secondary research questions:

1. Does preventing or treating human herpesviruses attenuate the risk of stroke?
2. Does the association between human herpesviruses and stroke vary by population characteristics (such as age and other common vascular risk factors)?

For peer review only - http://bmjopen.bmj.com/site/about/guidelines.xhtml

3. Does primary infection with or reactivation of human herpesviruses increase the risk of subtypes of stroke or transient ischaemic attack (TIA)?

These objectives will be addressed through a comprehensive review targeting all analytical epidemiological studies in humans of any age.

METHODS

This protocol follows the Preferred Reporting Items for Systematic Reviews and Meta Analyses Protocols (PRISMA-P) guidelines.[16]

Eligibility criteria

Study designs and characteristics: We will include studies using randomised controlled trials, cohort, case control, case crossover and self-controlled case series designs, reporting an effect estimate or the data that allow its calculation. We will exclude cross-sectional studies, ecological studies, case series, case reports and reviews, however relevant reviews will be flagged during the screening process and their references lists searched for potentially eligible studies. Studies from any time period, of any publication status, reported in any language and conducted in any geographical and healthcare setting (including in-patient, out-patient, primary care and community settings) will be considered.

Participants: Eligible studies will include human participants. Animal studies will not be included. No restrictions will be placed on studies according to the age and immunosuppression status of the participants.

For peer review only - http://bmjopen.bmj.com/site/about/guidelines.xhtml

Exposure: The exposures of interest are infection with or reactivation (first or subsequent) of the eight human herpesviruses: specifically, herpes simplex virus types 1 and 2, varicella zoster virus (VZV), Epstein-Barr virus (EBV), cytomegalovirus (CMV), human herpesvirus 6, human herpesvirus 7, and human herpesvirus 8. Studies involving an exposed participant group whose members self-report infection or reactivation with a human herpes virus, or who have a confirmed diagnosis, either through clinical or laboratory criteria, will be included in the review. We will also include vaccination against herpesviruses (e.g. zostavax vaccine) and treatment for herpesviruses (e.g. antivirals such as acyclovir) in order to investigate whether preventing or treating human herpesviruses attenuate the risk of stroke (a secondary research question).

Comparators: Eligible studies must include a comparator group who are unexposed, that is people (or person time for self-controlled case series designs) without herpesvirus infections or with latent herpesvirus infections.

Outcomes: Studies will be included in the review if the primary outcome was any stroke, clinically diagnosed or self-reported, and the patient's first ever or subsequent stroke. For studies meeting the inclusion criteria, we will additionally assess the following secondary outcomes: TIA (a transient episode of neurological dysfunction caused by focal brain, spinal cord, or retinal ischemia without acute infarction)[17] and subtypes of stroke (ischaemic versus haemorrhagic). Most strokes (approximately 85%)[2] are ischaemic (an episode of neurological dysfunction caused by focal, cerebral, spinal or retinal infarction)[17], compared to haemorrhagic (neurological dysfunction caused by a focal collection of blood within or on the surface of the brain).[17]

Eligibility criteria may be further developed, in an iterative process, after preliminary searches.

Information sources

For peer review only - http://bmjopen.bmj.com/site/about/guidelines.xhtml

For peer review only

The following databases will be searched for relevant articles, from dates of inception to January 2017; Cochrane Central Register of Controlled Trials (The Cochrane Library), Embase, Global Health, Medline, Scopus and Web of Science. Additional sources which will be searched include clinical trials registers (such as ClinicalTrials.gov) and grey literature including the New York Academy of Medicine Grey Literature Report (www.greylit.org) and the Electronic Theses Online Service through the British Library (http://ethos.bl.uk). PROSPERO will also be periodically checked for ongoing and completed systematic reviews concerning stroke and herpesviruses.

Search strategy

The search strategy will consist of searching medical subject heading (MeSH) terms and free text (in the title and abstract) for the concepts 'human herpesviruses' and 'stroke' (combined with the Boolean logic operator AND). The provisional search terms have been developed for the database MEDLINE and will be transcribed into appropriate search terms for the other information sources. The list of proposed search terms has been reviewed by all collaborators, including those with medical knowledge of the subject area, and necessary adjustments were made. The provisional search terms for MEDLINE are listed in the appendix.

We will review the reference lists of eligible articles and relevant reviews to identify additional papers not indexed in the databases searched.

Study records

Data management: Citations identified from the literature search will be downloaded into EndNote version X7.5 and duplicate records removed by one author.

Selection process: Two researchers (HF and CWG) will review all titles and abstracts in parallel to select studies for inclusion. To reduce the risk of missing potentially relevant studies, a deliberately

For peer review only - http://bmjopen.bmj.com/site/about/guidelines.xhtml

lenient approach will be adopted for this first level of screening. Both authors will then obtain full-text articles for studies deemed to potentially meet the review criteria. Reasons for rejection of articles during the full-text screening process will be noted, according to a hierarchical list (ineligible study design, wrong exposure, wrong outcome, insufficient information to calculate an effect estimate). Any discrepancies will be discussed by HF and CWG and consultation with a third reviewer (CM) will be carried out where necessary.

For peer review only

Data extraction: Information will be extracted from each study selected for review. Data extraction tables will be piloted by two authors (HF and CWG) for three studies and changes to the extraction tables made as required. Any discrepancies between the two authors will be discussed, and consultation with a third author (CM) carried out if required. Data will be extracted for each remaining study by a single author (HF). Consideration will be given to contacting corresponding authors for any missing information or clarification on unclear information, using a standard email template.

Data items

Data will be extracted using a standardised template. We will use the PICOS[18] (Population, Intervention, Comparator, Outcomes and Study design) framework, originally devised to formulate a research question, as a basis to develop data extraction criteria. As this is an aetiological study, “exposure” will replace “intervention” and “study characteristics” will replace “study design”. Data items on the following five domains will be extracted;

1. *Population:* characteristics of the study population (e.g. mean/median age, ethnic distribution, immune status), inclusion and exclusion criteria;
2. *Exposure:* definition and identification of human herpesvirus exposure, number of exposed subjects;

For peer review only - http://bmjopen.bmj.com/site/about/guidelines.xhtml

3. *Comparators:* definition and identification of unexposed individuals, number of unexposed subjects;
4. *Outcomes:* definition and identification of primary (stroke) and secondary outcomes (stroke subtypes or TIA), number of subjects with outcome;
5. *Study characteristics:* authors, publication year, setting/source of participants, design, methods of recruitment and sampling, period of study, length of follow-up time (if relevant), aims and objectives.

In terms of the study results, unadjusted and fully adjusted effect estimates for the association between herpesviruses and stroke will be recorded. Details of the confounders measured and adjusted for will also be noted. Results of any additional stratified analyses will also be recorded. Where possible, results from additional subgroup analyses with evidence regarding our non-primary objectives will also be recorded, for example the association between herpesviruses and the secondary outcomes (stroke subtype or TIA).

Outcomes and prioritization

The primary clinical outcome of interest is the first record of stroke following infection with or reactivation of a human herpesvirus. Where studies report several results for risk of stroke following herpesvirus exposure we will prioritise: stroke diagnosed objectively (e.g. through neuroimaging) or clinically, for example meeting the American Heart Association/American Stroke Association definition;[17] outcomes reported for the whole cohort (rather than subsets of the cohort, whose association between the herpesvirus and stroke may differ) and fully adjusted estimates of effect (rather than crude estimates). We will also extract data on the following secondary outcomes, where they are reported: TIA and subtypes of stroke (i.e. ischaemic versus haemorrhagic). Data extraction for these additional outcomes will be prioritised in the same way as the primary outcome. Studies in

For peer review only - http://bmjopen.bmj.com/site/about/guidelines.xhtml

For peer review only

which exposures were recorded prior to outcomes will be prioritized when considering the overall quality of included studies.

Risk of bias in individual studies

Two authors (HF and CWG) will independently evaluate the risk of bias in three studies, and any discrepancies will be discussed and our third reviewer (CM) consulted if necessary. HF will then carry out the risk of bias assessment for the remaining studies. We will consider a series of relevant areas of bias (or domains) for each individual study, in line with the Cochrane Collaborations risk of bias approach.[19-21] For observational studies, domains will include bias due to: 1) confounding; 2) selection of participants; and 3) differential and non-differential misclassification of variables (exposures, outcomes and covariates) 4) bias due to missing data. For randomised controlled trials, domains will include random sequence generation, allocation concealment, blinding, completeness of outcome data and selective reporting. Each domain will be classified as either 'high risk' (if criterion are inadequately addressed), 'low risk' (if criterion are adequately addressed) or 'unclear risk' (if information is insufficient to formulate a judgement). A summary risk of bias table will be produced, with an additional table briefly justifying each judgement included in the appendix.

Data synthesis and meta-bias(es)

We will use a narrative synthesis, in which studies are grouped by each specific herpesvirus exposure, to summarise the evidence for the association between the herpesvirus and our primary outcome (stroke). If there are sufficient data in the selected studies our narrative synthesis will also describe subgroup analyses, relevant to our secondary research questions. These include: 1) the effect of herpesviruses on stroke, according to whether patients were vaccinated (e.g. with the chickenpox vaccine or the herpes zoster vaccine) or received antiviral treatment against

For peer review only - http://bmjopen.bmj.com/site/about/guidelines.xhtml

herpesviruses; 2) the effect of herpesviruses on stroke for population characteristics, such as age strata and other common vascular risk factors; and 3) the effect of herpesviruses on the secondary outcomes TIA and stroke subtype (such as ischemic stroke or haemorrhagic stroke, timing of stroke, first or subsequent stroke).

If there are at least two eligible studies assessing the same herpesvirus as a risk factor for our primary (stroke) or secondary (TIA or stroke type) outcomes, which are sufficiently homogenous in terms of design, study population and outcome, we will consider conducting a meta-analysis to calculate a pooled effect estimate. The choice of whether to conduct a meta-analysis, and which model to adopt (fixed or random effects) will be guided by the level of statistical heterogeneity assessed using the Cochrane Q statistic and the I^2 statistic. An $I^2<50\%$ will be used as a threshold to indicate moderate heterogeneity and potential to use of a random effects model, if there is overall consistency in the direction of effect. We will investigate sources of heterogeneity by removing studies at high risk of bias and comparing summary estimates from different study-level methodological and clinical characteristics (such as stroke definition, study design and age of the study population), using meta-regression where appropriate. Publication bias will be considered using funnel plots. All of the statistical analysis will be performed using STATA version 14.0.

Confidence in cumulative evidence

The Grading of Recommendations, Assessment, Development and Evaluation (GRADE)[22] approach will be used to summarise the quality of cumulative evidence for each herpesvirus on our outcomes, stratified by exposure definition and population characteristics. In addition to the risk of bias domains outlined earlier, we will also assess inconsistency between studies, indirectness, imprecision of estimates and publication bias (using a funnel plot) as outlined in the GRADE

For peer review only - http://bmjopen.bmj.com/site/about/guidelines.xhtml

approach.[23] The strength of evidence will be categorised as 'high', 'moderate' or 'low/very low', with observational studies starting as low quality evidence, but upgraded to moderate or even high quality in the presence of factors that increase confidence in the estimated effect data (for example having a large magnitude of effect, evidence of a dose-response). These judgements will be presented in a 'Summary of Findings' table.

ETHICS AND DISSEMINATION

As this is a systematic review ethics approval is not required. The results will be submitted for peer-review publication and presented at national and international conferences. A lay and short summary will be disseminated on appropriate webpages. Important protocol amendments will be documented with a justification for deviating from the original protocol, and summarised in a protocol addendum and in the final published review.

References

1. Feigin VL, Forouzanfar MH, Krishnamurthi R, et al. Global and regional burden of stroke during 1990-2010: findings from the Global Burden of Disease Study 2010. Lancet 2014: 383(9913): 245-54.
2. Stroke Association. *State of the nation: Stroke Statistics*. 2016 [Cited 11 Jan 2017]; Available from: https://www.stroke.org.uk/resources/state-nation-stroke-statistics.
3. O'Donnell MJ, Xavier D, Liu L, et al. Risk factors for ischaemic and intracerebral haemorrhagic stroke in 22 countries (the INTERSTROKE study): a case-control study. The Lancet 376(9735): 112-123.
4. Esenwa CC and Elkind MS. Inflammatory risk factors, biomarkers and associated therapy in ischaemic stroke. Nature Reviews Neurology 2016: 12(10): 594-604.
5. Steiner I, Kennedy PG, and Pachner AR. The neurotropic herpes viruses: herpes simplex and varicella-zoster. Lancet Neurol 2007: 6(11): 1015-28.
6. Thomas SL, Minassian C, Ganesan V, et al. Chickenpox and Risk of Stroke: A Self-controlled Case Series Analysis. Clinical Infectious Diseases 2014: 58(1): 61-68.
7. Langan S, Minassian C, Smeeth L, and Thomas S. Risk of stroke following herpes zoster: a self-controlled case-series study. Journal of Investigative Dermatology 2014: 134: S39-S39.
8. Minassian C, Thomas SL, Smeeth L, et al. Acute Cardiovascular Events after Herpes Zoster: A Self-Controlled Case Series Analysis in Vaccinated and Unvaccinated Older Residents of the United States. Plos Medicine 2015: 12(12).

For peer review only - http://bmjopen.bmj.com/site/about/guidelines.xhtml

9. Kang JH, Ho JD, Chen YH, and Lin HC. Increased Risk of Stroke After a Herpes Zoster Attack A Population-Based Follow-Up Study. Stroke 2009: 40(11): 3443-3448.
10. Lin HC, Chien CW, and Ho JD. Herpes zoster ophthalmicus and the risk of stroke A population-based follow-up study. Neurology 2010: 74(10): 792-797.
11. Sundstrom K, Weibull CE, Soderberg-Lofdal K, et al. Incidence of herpes zoster and associated events including stroke-a population-based cohort study. Bmc Infectious Diseases 2015: 15.
12. Sreenivasan N, Basit S, Wohlfahrt J, et al. The Short- and Long-Term Risk of Stroke after Herpes Zoster - A Nationwide Population-Based Cohort Study. Plos One 2013: 8(7).
13. Yen YF, Chen M, Jen I, et al. Association of HIV and Opportunistic Infections with Incident Stroke: A Nationwide Population-based Cohort Study in Taiwan. J Acquir Immune Defic Syndr 2016.
14. Liu XC, Guan YM, Hou L, et al. The Short- and Long-Term Risk of Stroke after Herpes Zoster: A Meta-Analysis. Plos One 2016: 11(10).
15. Yang SY, Li HX, Yi XH, et al. Risk of Stroke in Patients with Herpes Zoster: A Systematic Review and Meta-Analysis. J Stroke Cerebrovasc Dis 2016.
16. Shamseer L, Moher D, Clarke M, et al. Preferred reporting items for systematic review and meta-analysis protocols (PRISMA-P) 2015: elaboration and explanation. BMJ 2015: 349: g7647.
17. Sacco RL, Kasner SE, Broderick JP, et al. An Updated Definition of Stroke for the 21st Century. A Statement for Healthcare Professionals From the American Heart Association/American Stroke Association 2013: 44(7): 2064-2089.
18. Higgins J and Green S. *Cochrane Handbook for Systematic Reviews of Interventions Version 5.1.0 [updated March 2011]. 6.4.2 Structure of a search strategy.* 2011 [Cited; Available from: www.handbook.cochrane.org.
19. Sterne JA, Hernan MA, Reeves BC, et al. ROBINS-I: a tool for assessing risk of bias in non-randomised studies of interventions. BMJ 2016: 355: i4919.
20. Higgins JP, Altman DG, Gotzsche PC, et al. The Cochrane Collaboration's tool for assessing risk of bias in randomised trials. BMJ 2011: 343: d5928.
21. Sterne JA, Hernán MA, Reeves BC, et al. ROBINS-I: a tool for assessing risk of bias in non-randomised studies of interventions. BMJ 2016: 355.
22. Atkins D, Best D, Briss PA, et al. Grading quality of evidence and strength of recommendations. British Medical Journal 2004: 328(7454): 1490-1494.
23. Balshem H, Helfand M, Schunemann HJ, et al. GRADE guidelines: 3. Rating the quality of evidence. Journal of Clinical Epidemiology 2011: 64(4): 401-406.

Authors' contributions

HF contributed to the design of the study, drafted the methods and analysis and revised the protocol following author comments; LB contributed to the design of the study and revised the paper critically; JB contributed to the conception and design of the study and revised the paper critically; MB contributed to the design of the study and revised the paper critically; SML contributed to the design of the study and revised the paper critically; CM contributed to the design of the study and revised the paper critically; LS contributed to the conception and design of the study and revised the paper critically; SLT contributed to the design of the study and revised the paper critically; CWG

For peer review only - http://bmjopen.bmj.com/site/about/guidelines.xhtml

conceived and designed the study, drafted the introduction and made critical comments on the protocol. All authors approved the final version of the protocol.

Funding statement

Funded by a Wellcome Trust Intermediate Clinical Fellowship to Dr C. Warren-Gash (201440/Z/16/Z). The funder played no role in developing the protocol. JB receives funding from the NIHR UCL/UCLH . BRC. SML is funded by a Wellcome Trust Senior Research Fellowship in Clinical Science (205039/Z/16/Z). CM is supported by a Wellcome Trust Senior Fellowship in Clinical Science (to LS, grant number: 098504/Z/12/Z). LB is supported by NIHR Clinical Lectureship funding. LS is supported by a Senior Clinical Fellowship from Wellcome. MMB's Chair in Stroke Medicine is supported by the Reta Lila Weston Trust for Medical Research. Part of this work was undertaken at University College London, which received a proportion of funding from the UK Department of Health's National Institute for Health Research Biomedical Research Centres funding scheme.

Competing interests statement

HF - none; LB – none; JB – none; MB - none; SML – none; CM – none; LS – none; SLT – none; CWG – none.

For peer review only

For peer review only - http://bmjopen.bmj.com/site/about/guidelines.xhtml

1 exp Herpes simplex
2 exp Herpes simplex virus vaccines
3 exp encephalitis, herpes simplex
4 exp Herpesvirus 1, Human
5 cold sore$.ti,ab.
6 exp Herpesvirus 2, Human
7 (genit$ herpes$ or genit$ sores).ti,ab.
8 exp Chickenpox
9 exp Chickenpox vaccine
10 exp Herpes zoster
11 exp Neuralgia, postherpetic
12 exp Herpesvirus 3, Human
13 exp Encephalitis, varicella zoster
14 (varicella or chickenpox or chicken pox or shingles or VZV or zoster).ti,ab.
15 exp Cytomegalovirus
16 exp Cytomegalovirus vaccines
17 exp Cytomegalovirus infections
18 (CMV or cytomegalovirus).ti,ab.
19 exp Herpesvirus 6, Human
20 Roseolovirus Infections/
21 Exanthema Subitum/
22 (B lymphotropic virus$ or roseola or sixth disease or exanthema subitum or exanthem criticum or Roseolovirus or pseudorubella or three?day fever).ti,ab.
23 exp Herpesvirus 7, Human
24 exp Epstein-Barr virus infections
25 exp Epstein-Barr virus
26 exp Herpesvirus 4, Human
27 (EBV or epstein-barr or burkitt adj5 lymphoma$ or glandular fever or infectious mono$ or mononucleosis or hair$ leukoplak$ or OHL).ti,a
28 exp Herpesvirus 8, Human
29 Sarcoma, Kaposi/
30 Lymphoma, Primary Effusion/
31 (kaposi$ sarcoma$ or Primary effusion adj2 lymphoma$ or body cavity adj2 lymphoma$).ti,ab.
32 ((HHV adj1 ("1" or "2" or "3" or "4" or "5" or "6" or "7" or "8")) or (HHV?1 or HHV?2 or HHV?3 or HHV?4 or HHV?5 or HHV?6 or HHV?7 or HHV?8)).ti,ab.
33 (HSV?1 or HSV 1 or HSV?2 or HSV 2).ti,ab.
34 herpes$.ti, ab.
35 exp acyclovir
36 ganciclovir/ or foscarnet/ or Idoxuridine/ or Trifluridine/
37 (ac?clovir or Zovirax or valac?clovir or valtrex or famc?clovir or famvir or penc?clovir or ganc?clovir or cidofovir or foscarnet$ or valganc?clovir or lubocavir or brivudin or Docosanol or Sorivudine or Idoxuridine or Trifluridine).ti,ab
38 or 1/37
39 exp stroke/
40 exp "Intracranial Embolism and Thrombosis"/
41 exp intracranial hemorrhages/
42 aneurysm, ruptured/ and exp brain/
43 Ischemic Attack, Transient/
44 (stroke or cva or (cerebrovasc$ AND (disease or event or accident or attack or injury))).ti,ab.
45 ((brain$ or cerebr$ or cerebell$ or cortical or vertebrobasilar or hemispher$ or intracran$ or intracerebral or infratentorial or supratentorial or MCA or anterior circulation or posterior circulation or basal ganglia) adj5 (isch?emi$ or infarct$)).ti,ab.
46 (((lacunar or cortical) adj5 infarct$)).ti,ab.
47 ((intracran$ or intracerebral) adj3 (thrombo$ or thrombus$ or embol$)).ti,ab.
48 SAH.ti,ab.
49 ((brain$ or cerebr$ or cerebell$ or intracerebral or intracran$ or parenchymal or intraventricular or infratentorial or supratentorial or basal gangli$ or subarachnoid or putaminal or putamen or posterior fossa) adj5 (haemorrhage$ or hemorrhage$ or bleed$ or rupture$ adj3 aneurysm)).ti,ab.
50 ((tia$1 or transi$ adj3 (isch?emia$ attack or brain isch?emia$ or cerebral isch?emia$ or CVA$ or cerebral vasc$ or cerebrovasc$)).ti,ab.
51 or 39/50
52 38 and 51

For peer review only

TRIAL MODE - a valid license will remove this message. See the keywords property of this PDF for more information.

PRISMA-P (Preferred Reporting Items for Systematic review and Meta-Analysis Protocols) 2015 checklist: recommended items to address in a systematic review protocol*

Section and topic	Item No	Checklist item		
**ADMINISTRATIVE INFORMATION**				
Title:				
Identification	1a	Identify the report as a protocol of a systematic review	✓	P1
Update	1b	If the protocol is for an update of a previous systematic review, identify as such	-	-
Registration	2	If registered, provide the name of the registry (such as PROSPERO) and registration number	✓	P2
Authors:				
Contact	3a	Provide name, institutional affiliation, e-mail address of all protocol authors; provide physical mailing address of corresponding author	✓	P1
Contributions	3b	Describe contributions of protocol authors and identify the guarantor of the review	✓	P14
Amendments	4	If the protocol represents an amendment of a previously completed or published protocol, identify as such and list changes; otherwise, state plan for documenting important protocol amendments	-	-
Support:				
Sources	5a	Indicate sources of financial or other support for the review	✓	P15
Sponsor	5b	Provide name for the review funder and/or sponsor	✓	P15
Role of sponsor or funder	5c	Describe roles of funder(s), sponsor(s), and/or institution(s), if any, in developing the protocol	✓	P15
**INTRODUCTION**				
Rationale	6	Describe the rationale for the review in the context of what is already known	✓	P4/5
Objectives	7	Provide an explicit statement of the question(s) the review will address with reference to participants, interventions, comparators, and outcomes (PICO)	✓	P5
**METHODS**				
Eligibility criteria	8	Specify the study characteristics (such as PICO, study design, setting, time frame) and report characteristics (such as years considered, language, publication status) to be used as criteria for eligibility for the review	✓	P6/7
Information sources	9	Describe all intended information sources (such as electronic databases, contact with study authors, trial registers or other grey literature sources) with planned dates of coverage	✓	P7/8

For peer review only

Search strategy	10	Present draft of search strategy to be used for at least one electronic database, including planned limits, such that it could be repeated	✓	P8
Study records:				
Data management	11a	Describe the mechanism(s) that will be used to manage records and data throughout the review	✓	P8
Selection process	11b	State the process that will be used for selecting studies (such as two independent reviewers) through each phase of the review (that is, screening, eligibility and inclusion in meta-analysis)	✓	P8/9
Data collection process	11c	Describe planned method of extracting data from reports (such as piloting forms, done independently, in duplicate), any processes for obtaining and confirming data from investigators	✓	P9
Data items	12	List and define all variables for which data will be sought (such as PICO items, funding sources), any pre-planned data assumptions and simplifications	✓	P9/10
Outcomes and prioritization	13	List and define all outcomes for which data will be sought, including prioritization of main and additional outcomes, with rationale	✓	P10
Risk of bias in individual studies	14	Describe anticipated methods for assessing risk of bias of individual studies, including whether this will be done at the outcome or study level, or both; state how this information will be used in data synthesis	✓	P11
Data synthesis	15a	Describe criteria under which study data will be quantitatively synthesised	✓	P11/12
	15b	If data are appropriate for quantitative synthesis, describe planned summary measures, methods of handling data and methods of combining data from studies, including any planned exploration of consistency (such as I^2, Kendall's τ)	✓	P11/12
	15c	Describe any proposed additional analyses (such as sensitivity or subgroup analyses, meta-regression)	✓	P11/12
	15d	If quantitative synthesis is not appropriate, describe the type of summary planned	✓	P11/12
Meta-bias(es)	16	Specify any planned assessment of meta-bias(es) (such as publication bias across studies, selective reporting within studies)	✓	P11/12
Confidence in cumulative evidence	17	Describe how the strength of the body of evidence will be assessed (such as GRADE)	✓	P12/13

*** It is strongly recommended that this checklist be read in conjunction with the PRISMA-P Explanation and Elaboration (cite when available) for important clarification on the items. Amendments to a review protocol should be tracked and dated. The copyright for PRISMA-P (including checklist) is held by the PRISMA-P Group and is distributed under a Creative Commons Attribution Licence 4.0.**

From: Shamseer L, Moher D, Clarke M, Ghersi D, Liberati A, Petticrew M, Shekelle P, Stewart L, PRISMA-P Group. Preferred reporting items for systematic review and meta-analysis protocols (PRISMA-P) 2015: elaboration and explanation. BMJ. 2015 Jan 2;349(jan02 1):g7647.

For peer review only
